# RACIAL TRAUMA IN EMERGING ADULTS RAISED BY GRANDPARENTS: PROTECTING AGAINST DISCRIMINATION

**DOI:** 10.1093/geroni/igac059.1034

**Published:** 2022-12-20

**Authors:** Acacia Lopez, Rachel Scott, Marin Olson, Danielle Nadorff

**Affiliations:** Mississippi State University, Starkville, Mississippi, United States; Mississippi State University, Starkville, Mississippi, United States; Mississippi State University, Mississippi State, Mississippi, United States; Mississippi State University, Mississippi State, Mississippi, United States

## Abstract

Experiences of racial trauma are linked with psychopathology, but a strong ethnic identity may serve as a protective factor. Grandparents primarily influence the development of ethnic identity, and BIPOC children are increasingly being raised by grandparents. Secure attachments influence stronger ethnic identities, yet custodial grandchildren are at higher risk of disrupted attachments. The current study investigated whether ethnic identity would mediate the relation between attachment and racial trauma symptoms in emerging adults previously raised by their grandparents and their peers (N = 370; 33% raised by grandparents; 25.6% non-white), with race as a moderator. Across all races, there were group differences in symptoms of racial trauma, with those not raised by grandparents experiencing a direct effect of race on ethnic identity. Attachment was a significant predictor of trauma symptoms of discrimination, moderated by race. Implications may provide support for clinical interventions addressing attachment and ethnic identity to decrease trauma symptoms.

